# Evaluation of Intestinal Phosphate Binding to Improve the Safety Profile of Oral Sodium Phosphate Bowel Cleansing

**DOI:** 10.1371/journal.pone.0116590

**Published:** 2015-03-19

**Authors:** Stef Robijn, Benjamin A. Vervaet, Patrick C. D’Haese, Anja Verhulst

**Affiliations:** Laboratory of Pathophysiology, University of Antwerp, Antwerp, Belgium; University of Louisville, UNITED STATES

## Abstract

Prior to colonoscopy, bowel cleansing is performed for which frequently oral sodium phosphate (OSP) is used. OSP results in significant hyperphosphatemia and cases of acute kidney injury (AKI) referred to as acute phosphate nephropathy (APN; characterized by nephrocalcinosis) are reported after OSP use, which led to a US-FDA warning. To improve the safety profile of OSP, it was evaluated whether the side-effects of OSP could be prevented with intestinal phosphate binders. Hereto a Wistar rat model of APN was developed. OSP administration (2 times 1.2 g phosphate by gavage) with a 12h time interval induced bowel cleansing (severe diarrhea) and significant hyperphosphatemia (21.79 ± 5.07 mg/dl 6h after the second OSP dose versus 8.44 ± 0.97 mg/dl at baseline). Concomitantly, serum PTH levels increased fivefold and FGF-23 levels showed a threefold increase, while serum calcium levels significantly decreased from 11.29 ± 0.53 mg/dl at baseline to 8.68 ± 0.79 mg/dl after OSP. OSP administration induced weaker NaPi-2a staining along the apical proximal tubular membrane. APN was induced: serum creatinine increased (1.5 times baseline) and nephrocalcinosis developed (increased renal calcium and phosphate content and calcium phosphate deposits on Von Kossa stained kidney sections). Intestinal phosphate binding (lanthanum carbonate or aluminum hydroxide) was not able to attenuate the OSP induced side-effects. In conclusion, a clinically relevant rat model of APN was developed. Animals showed increased serum phosphate levels similar to those reported in humans and developed APN. No evidence was found for an improved safety profile of OSP by using intestinal phosphate binders.

## Introduction

Colonoscopy is the standard procedure for diagnostic evaluation of the colon, with more than 14 million examinations performed annually in the United States alone. [1] As its efficacy largely depends on the degree to which fecal material is removed from the colon, bowel cleansing is carried out for which frequently oral sodium phosphate (OSP) solutions or tablets are used. OSP increases the osmolality of the intestinal lumen, thereby retaining fluid and inducing peristalsis and colonic evacuation. [2,3] The major advantage of OSP is the relatively low volume required for efficient bowel cleansing, generally resulting in a higher patient compliance and better efficacy compared to other agents such as polyethylene glycol (PEG). [4–11] For years, a standard OSP procedure consisted of two 45 ml (or 20 tablets) doses which had to be taken with a time interval of 10–12 hours (usually the evening before and the morning of the colonoscopy). As each dose contained a mixture of NaH_2_PO_4_ and Na_2_HPO_4_ equivalent to ∼6 g phosphorus (∼18 g phosphate), it far exceeded the normal daily intake of ∼1 g phosphorus. [12]

Since the case report of Desmeules *et al*. [13] in 2003, an increasing number of cases [14–26] are described in which patients develop (in some cases irreversible) acute kidney injury (AKI) after the use of OSP. Renal biopsy findings include calcium phosphate (CaP) deposits in the distal tubules and collecting ducts, often accompanied by tubular atrophy and mild to moderate interstitial fibrosis, which led to the term “acute phosphate nephropathy” (APN). [12,14,27] This condition can be attributed to the acute OSP load which leads to significantly increased serum phosphate levels that stimulate PTH secretion which, in turn, leads to endocytosis of the NaPi-2a/c co-transporters from the brush border of the proximal tubules. This endocytosis inhibits renal phosphate reabsorption and, hence, results in significant urinary phosphate wasting. [28–30] After OSP administration, serum phosphate levels double compared to baseline and urinary phosphate excretion increases eightfold, which may result in intratubular CaP crystal formation and retention. [12,29,31,32]

Following its initial use, the recommended OSP dose was reduced (a 45 ml or 20 tablets dose followed by a 30 ml or 12 tablets dose), providing equally effective bowel cleansing with less pronounced, however still significant, hyperphosphatemia. [10,33] In 2008, however, the US Food and Drug Administration issued a safety alert stating that OSP for bowel cleansing should only be available by prescription which eventually caused a discontinuation of many (over-the-counter) OSP products. Currently, OSP remains available in tablet form by prescription only. [3,12,27,34–36]

As OSP generally results in a higher patient compliance and better efficacy compared to PEG, an important therapeutic approach could consist of the prevention of phosphate absorption to improve the safety profile of OSP. [10,27] In this context, the potential use of lanthanum carbonate, an efficient non-calcium containing phosphate binder with very low systemic absorption, used to treat hyperphosphatemia in dialysis patients is worthy of consideration. [37] Aluminum-based phosphate binders are also highly effective. Despite their association with cognitive disturbances, osteomalacia and anemia due to aluminum accumulation over time in patients with end-stage renal disease (aluminum is excreted exclusively by the kidney), they still remain in use either in combination with other phosphate binding agents or for acute control of hyperphosphatemia where risks of accumulation are minimal. [38,39] Its elimination pathway implies that there is no concern about aluminum accumulation in subjects with normal renal function.

In this study, a model of APN was developed in the Wistar rat. As well as the known acute effects of OSP (hyperphosphatemia, hypocalcemia and APN), the effects of OSP on serum PTH and FGF-23 levels and on the expression of NaPi co-transporter proteins in the kidney (NaPi-2a) were investigated. Finally, it was determined whether OSP induced side-effects could be prevented by intestinal phosphate binding using either lanthanum carbonate or aluminum hydroxide.

## Materials and Methods

### Optimization of the composition of the OSP dose

In a pilot experiment, 20 male Wistar rats (∼300 g; Charles River) were randomly assigned to four groups (n = 5 per group) receiving a single OSP dose corresponding to (i) 0.4 g, (ii) 0.45 g, (iii) 0.5 g and (iv) 0.55 g phosphorus (corresponding to 1.2 g; 1.35 g; 1.5 g and 1.65 g phosphate, respectively) by gavage. At different time points (baseline, 1 hour, 3 hours, 6 hours and 24 hours after the OSP dose) blood was drawn from the tail vein in restrained, conscious animals for the determination of serum phosphate. In a second phase, the optimized OSP dose (corresponding to 0.4 g phosphorus; 1.2 g phosphate) was administered to 6 male Wistar rats (∼360 g; Charles River) by gavage based on the standard OSP dosing regimen (2 OSP doses with a time interval of 12 hours). Animals were fasted during the study period, but had access to tap water *ad libitum*. At different time points (baseline, 12 hours after the first OSP dose and 3 hours, 6 hours, 24 hours and 48 hours after the second dose) blood was drawn from the tail vein for the determination of serum phosphate.

The composition of the OSP dose was based on the therapeutically used Fleet Phospho-soda [2,12,40] and contained Na_2_HPO_4_. 2 H_2_O (Analar) and NaH_2_PO_4_. H_2_O (Merck) in a weight ratio of ∼1:2.3.

### Evaluation of the rat model of APN and effect of intestinal phosphate binding

30 male Wistar rats (∼380 g; Charles River) were randomly assigned to five experimental groups (n = 6 per group): (i) a group receiving 1000 mg lanthanum carbonate (suspended in 0.1% carboxymethylcellulose; CMC) followed by OSP corresponding to 0.4 g phosphorus (1.2 g phosphate; 0.3x molar lanthanum/phosphate ratio), (ii) a group receiving 289.5 mg aluminum hydroxide (suspended in 0.1% CMC) followed by the same OSP dose (0.3x molar aluminum/phosphate ratio), (iii) a group receiving 1000 mg aluminum hydroxide (suspended in 0.1% CMC) followed by the same OSP dose (1x molar aluminum/phosphate ratio), (iv) a group receiving vehicle (0.1% CMC) followed by the same OSP dose and (v) a control group receiving vehicle followed by water by gavage. Based on the standard OSP dosing regimen, two OSP doses (together with lanthanum carbonate, aluminum hydroxide or vehicle) were administered with a time interval of 12 hours. Animals were fasted during the study period, but had access to tap water *ad libitum*.

Lanthanum carbonate, aluminum hydroxide (or vehicle) and OSP (or water) were administered through separate gavages to avoid phosphate binding prior to arrival in the intestine which would not reflect actual intestinal complexation.

At different time points (baseline, 12 hours after the first OSP dose and 3 hours after the second OSP dose) blood was drawn from the tail vein in restrained, conscious animals for the determination of a series of serum parameters. At sacrifice (6 hours after the second OSP dose), animals were anaesthetized with 30 mg/kg sodium pentobarbital (Nembutal; Ceva Santé Animale) via intravenous injection, opened through a midline incision and exsanguinated through the aorta abdominalis. The left kidney and intestinal tract were removed and slices of renal tissue were fixed in neutral buffered formalin (FNB) for 4 hours, rinsed with 70% isopropanol and embedded in paraffin for (immuno-) histology or digested in 65% nitric acid for chemical analysis. The content of the entire colon and terminal 5 cm of the ileum was collected to examine bowel cleansing.

### Ethics statement

Experimental procedures were conducted according to the National Institutes of Health Guide for the Care and Use of Laboratory Animals and approved by the University of Antwerp Ethics Committee (Permit number ED2011-68).

### Serum biochemistry

Serum phosphate (mg/dl) was measured using the Ecoline Phosphate FS assay (DiaSys). Serum calcium (mg/dl) was determined by flame atomic absorption spectrometry (FAAS; Perkin-Elmer). Serum creatinine (mg/dl) was measured using the kinetic alkaline picrate (Jaffe) reaction. Serum intact PTH (Immutopics) (pg/ml) and intact FGF-23 (Kainos Laboratories) (pg/ml) were both determined using ELISA kits.

### Bowel cleansing

Besides visual observation of diarrhea, bowel cleansing was examined by collecting the bowel content of the entire colon as well as the terminal 5 cm of the ileum in a 15 ml centrifuge tube during sacrifice. Water was added up to a volume of 14 ml and content was vigorously mixed. Subsequently, the tubes were centrifuged at 3000 rpm (1620 g) for 45 minutes and the percentage of visible insoluble bowel content (pellet; 14 ml equals 100%) was used as a measure of bowel cleansing. [40]

### Renal CaP calcifications

Renal calcifications were visualized by Von Kossa staining. Deparaffinized 4 μm tissue sections were incubated in 5% silver nitrate for 45 minutes. Slides were rinsed in water, incubated in 1% pyrogallol for 3 minutes, rinsed with water, fixed in 5% sodium thiosulfate for 1 minute and counterstained with hematoxylin/eosin. In each section CaP deposits were quantified histomorphometrically using Axiovision 4.5 software (Carl Zeiss). Renal calcification is expressed as area% and was calculated as the ratio of Von Kossa positive (pixel²) on renal tissue (pixel²) area.

Renal crystal content was also quantitatively assessed by measuring calcium and phosphate content in renal tissue digestion solutions. Briefly, transversal slices of renal tissue were weighed after removal, digested in 65% nitric acid at 60°C overnight and diluted in 0.1% lanthanum nitrate to eliminate chemical interference during subsequent spectrometric analysis. In this solution total calcium content was measured by FAAS (Perkin-Elmer) and total phosphate content using the Ecoline Phosphate FS assay (DiaSys).

### Immunohistochemistry

Immunohistochemical staining was performed on FNB-fixed, deparaffinized renal sections for NaPi-2a. Heat-induced epitope retrieval was performed by microwaving the sections (2x 5 min) in 0.1 M citrate buffer (pH 6.0). The tissue sections were then blocked with normal goat serum (20% in TSB with 1% Triton X-100) for 20 min and incubated overnight with polyclonal rabbit anti-human NaPi-2a (1:40; Novus Biologicals; NBP2-13328). Biotinylated goat anti-rabbit (Vector Laboratories) was used as secondary antibody. Avidin/biotinylated peroxidase complex (VECTASTAIN ABC KIT, Vector Laboratories) was added as signal amplifier and 3-Amino-9-ethylcarbazole (AEC, Sigma-Aldrich) was used as substrate. The sections were counterstained with hematoxylin. Sections in which the primary antibody was omitted were used as negative controls.

### Statistical analysis

Data are either presented as mean ± standard deviation, individual values or as median (range). Differences between multiple time points for each group were determined by the Friedman test, followed by a Wilcoxon-signed-rank test. Comparisons between the groups for each time point were assessed using a Kruskal–Wallis test, followed by a Mann–Whitney-U test. Values of p<0.05 were considered significant. Spearman correlations were made between the renal calcium and phosphate content and the histomorphometrical calcification score and between the serum parameters (FGF-23, PTH, calcium and phosphate). Statistics were performed with SPSS Statistics 20.0.

## Results

### Optimization of the composition of the OSP dose

Administration of a single OSP dose resulted in significantly increased serum phosphate levels for all tested doses (0.4 g, 0.45 g, 0.5 g and 0.55 g phosphorus) and induced diarrhea in all animals, reflecting efficient bowel cleansing. However, because of the mortality in the groups receiving 0.5 g and 0.55 g phosphorus (20% and 80% respectively), it was decided to use the lowest dose (0.4 g phosphorus) in the second phase of the optimization study.

Two OSP doses (corresponding each to 0.4 g phosphorus) with a 12 hours’ time interval induced diarrhea in all animals and resulted in significantly increased serum phosphate concentrations with the highest levels (nearly two times baseline and thus resembling the clinical situation in humans receiving OSP) [31,32] occurring 6 hours after the second dose (14.8 ± 3.1 vs 9.2 ± 0.6 mg/dl at baseline) and without mortality. This OSP dosing regimen was further evaluated.

### Evaluation of the rat model of APN

#### Acute serum effects of OSP

**Fig 1:**
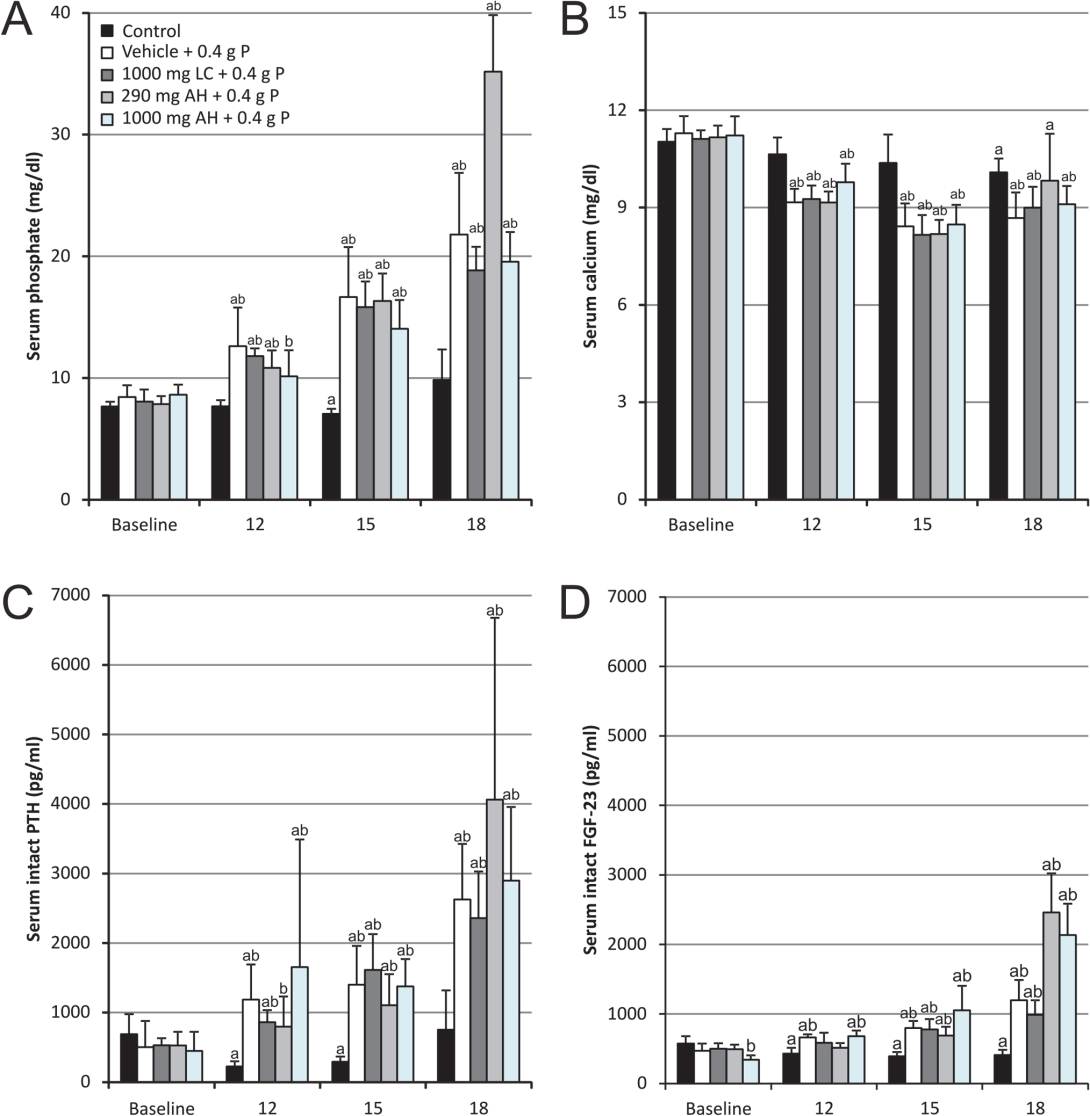
Serum (A) phosphate, (B) calcium, (C) intact PTH and (D) intact FGF-23 levels. Data are presented as mean ± standard deviation. (^a^ P<0.05 vs baseline; ^b^ P<0.05 vs control) (12: 12 hours after the first OSP dose; 15: 3 hours after the second OSP dose and 18: 6 hours after the second OSP dose; n = 6 per group; statistical tests: Mann–Whitney-U and Wilcoxon-signed-rank).

Administration of two OSP doses (corresponding each to 0.4 g phosphorus or 1.2 g phosphate) with a 12 hours’ time interval resulted in transiently increased serum phosphate levels compared to control animals (Fig. 1A). Serum phosphate levels peaked at 6 hours after the administration of the second OSP dose and increased up to more than twofold, i.e. 21.79 ± 5.07 mg/dl versus 8.44 ± 0.97 mg/dl at baseline. Concomitantly, serum calcium significantly decreased from 11.29 ± 0.53 mg/dl at baseline to 8.68 ± 0.79 mg/dl after OSP (Fig. 1B). Serum phosphate and calcium negatively correlated with each other (r = -0.75, p<0.01; Table 1). Together with the acute changes in serum phosphate and calcium levels, serum (intact) PTH (Fig. 1C) and (intact) FGF-23 (Fig. 1D) levels also significantly increased already at 12 hours after the first OSP dose. The increase in serum PTH concentration was more pronounced than that of FGF-23. While for PTH a fivefold increase versus baseline was seen, FGF-23 levels showed a threefold increase after OSP administration. Both serum FGF-23 and serum PTH strongly correlated with serum phosphate (r = 0.85 and r = 0.80 respectively, p<0.01; Table 1), whereas for serum calcium levels there was a negative correlation with PTH as well as FGF-23 (r = -0.55 and r = -0.69 respectively, p<0.01; Table 1).

**Table 1:** Correlations between serum parameters in animals receiving OSP.

Spearman correlation coefficient (r)	Serum phosphate	Serum calcium	Serum PTH	Serum FGF-23
Serum phosphate	X	-0.75**	0.80**	0.85**
Serum calcium	-0.75**	X	-0.55**	-0.69**
Serum PTH	0.80**	-0.55**	X	0.79**
Serum FGF-23	0.85**	-0.69**	0.79**	X

** p<0.01

#### Acute phosphate nephropathy

**Fig 2:**
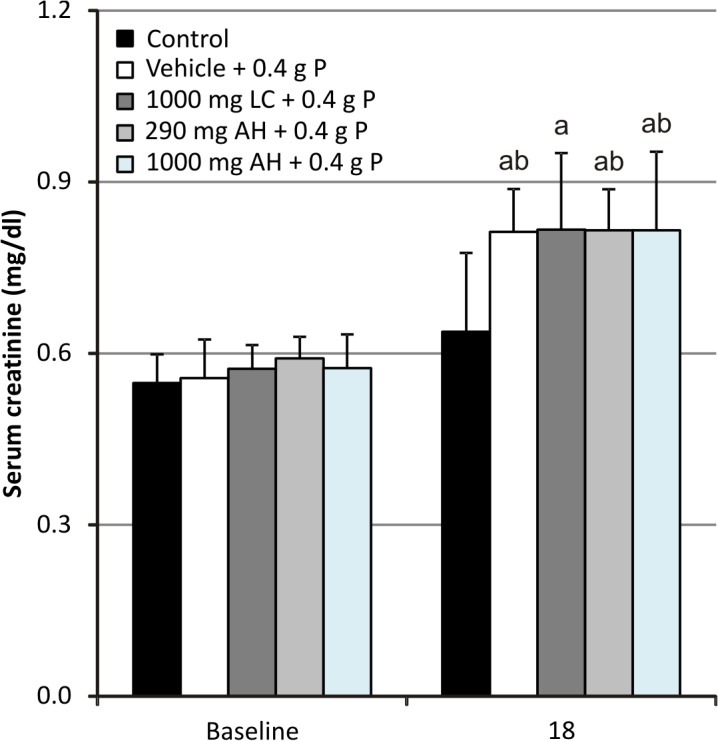
Serum creatinine levels. Data are presented as mean ± standard deviation. (^a^ P<0.05 vs baseline; ^b^ P<0.05 vs control; 18: 6 hours after the second OSP dose; n = 6 per group; statistical tests: Mann–Whitney-U and Wilcoxon-signed-rank).

Serum creatinine concentrations (Fig. 2) significantly increased to 1.5 times baseline after OSP administration, indicating the development of AKI (KDIGO). [41]

**Fig 3:**
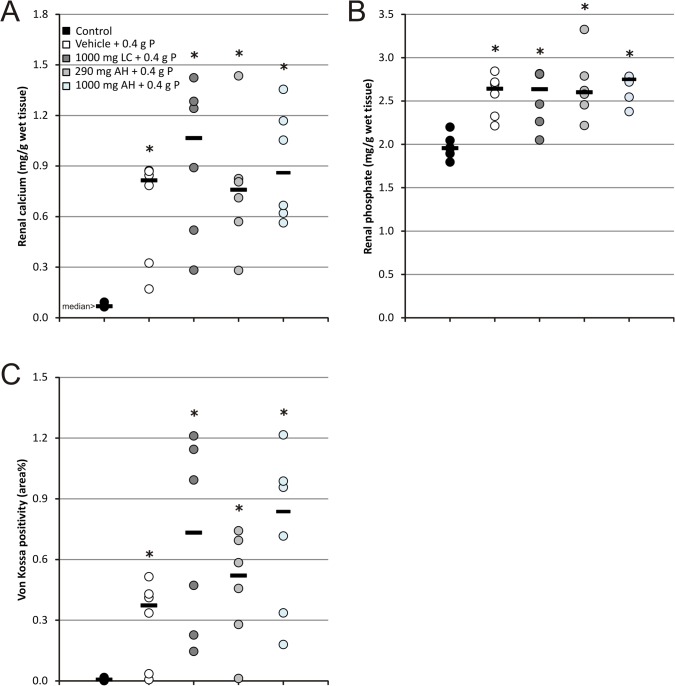
(A) Renal calcium content, (B) renal phosphate content and (C) histomorphometrical quantification of Von Kossa stained kidney sections. Data are presented as individual values (circles) and median (horizontal bars). (* P<0.05 vs control; n = 6 per group; statistical test: Mann–Whitney-U).

**Fig 4:**
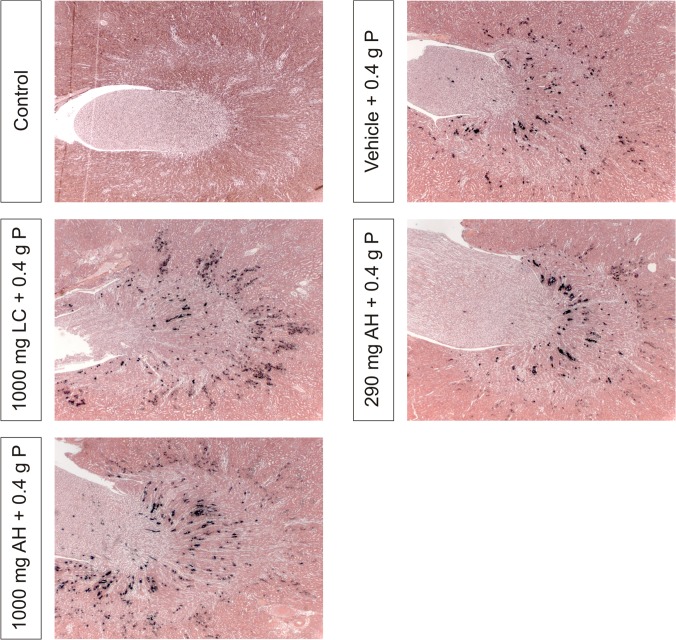
Von Kossa stained renal sections, representative of each treatment group. Magnification x31.25.

Renal calcium content (Fig. 3A) was significantly higher in animals receiving OSP as compared to control animals with a median of 0.815 mg/g wet renal tissue (range: 0.170–0.868 mg/g) compared to 0.068 mg/g wet renal tissue (range: 0.064–0.092 mg/g). A similar pattern was observed for the renal phosphate content (Fig. 3B) with a median of 2.64 mg/g wet renal tissue (range: 2.21–2.84 mg/g) in animals receiving OSP compared to 1.96 mg/g wet renal tissue (range: 1.79–2.20 mg/g) in controls. Histomorphometric quantification of Von Kossa stained kidney sections (Fig. 3C) confirmed these results. Renal calcium and phosphate concentrations strongly correlated with each other (r = 0.80, p<0.01) as well as with the area% Von Kossa positivity (r = 0.82 and r = 0.74 respectively, p<0.01). The calcifications were mainly confined to the medulla (Fig. 4).

#### Expression of renal NaPi co-transporters

**Fig 5:**
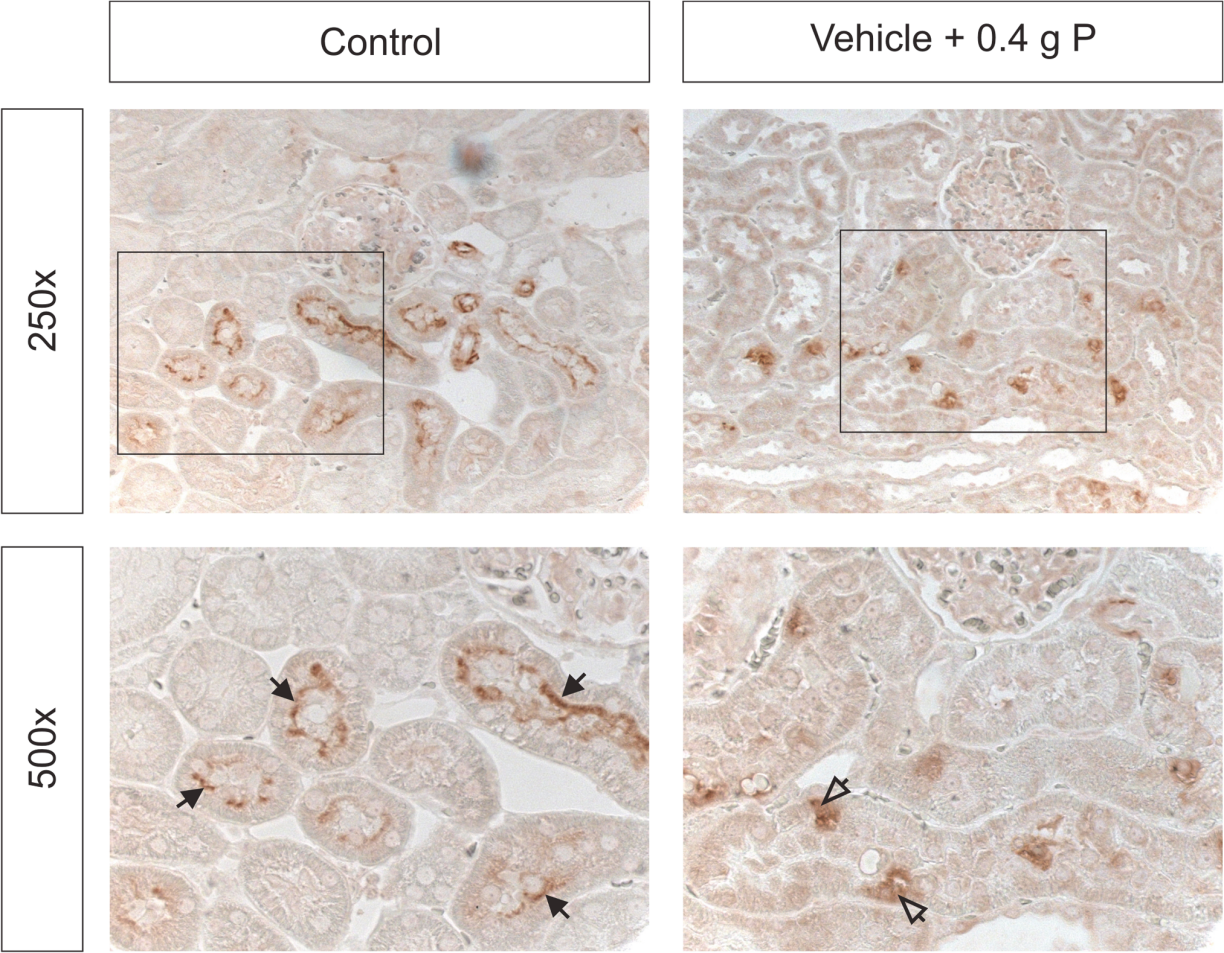
Immunohistochemical analysis of the apical (closed arrows) and intracellular (open arrows) NaPi-2a expression in the kidney.

NaPi-2a was clearly localized to the apical membrane of proximal tubules in control animals. In animals receiving OSP, NaPi-2a staining was weaker along the apical membrane and appeared more intracellular (Fig. 5).

#### Bowel cleansing

**Fig 6:**
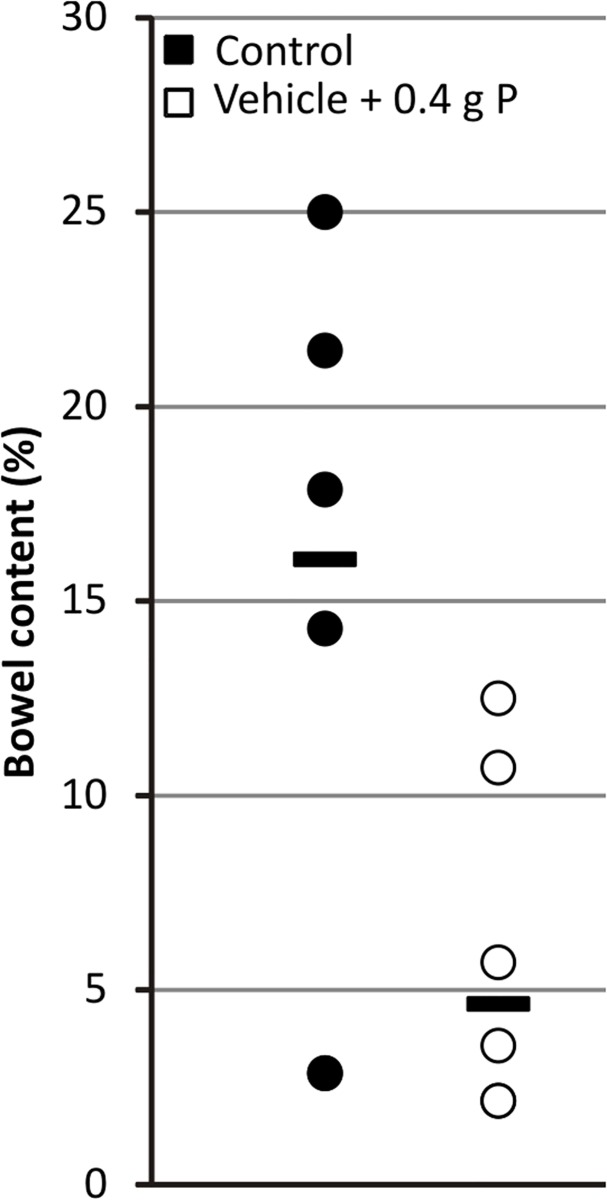
Effect of OSP on bowel content. Data are presented as individual values (circles) and median (horizontal bars). (n = 6 per group; statistical test: Mann–Whitney-U).

OSP administration induced diarrhea in all animals. The amount of insoluble bowel content of the entire colon and terminal 5 cm of the ileum (used as a measure of bowel cleansing) decreased from a median of 16.1% (range: 2.9–25.0%) in control animals to 4.6% (range: 2.1–12.5%) in animals receiving OSP (Fig. 6).

### Effect of intestinal phosphate binding

Neither phosphate binder treatment was able to attenuate the serological effects of OSP (serum phosphate, calcium, PTH and FGF-23 levels; Fig. 1) and as a result did not have any effect on the development of APN (Figs. 2, 3 and 4).

## Discussion

It is presumed that the incidence of APN is highly underestimated because many cases would remain undiagnosed (clinically silent) when the amount of affected renal tubules is limited. [24,29] As OSP generally results in a higher patient compliance and better efficacy compared to PEG, an important therapeutic approach could consist of the prevention of phosphate absorption to improve its safety profile, [10,27] as adequate hydration only may not be able to prevent hyperphosphatemia and precipitation of CaP crystals. [42,43] Therefore, in this study, it was investigated whether the acute side-effects of OSP administration (hyperphosphatemia, hypocalcemia and APN) could be prevented by inhibition of intestinal phosphate absorption with two therapeutically used phosphate binding agents, i.e. lanthanum carbonate or aluminum hydroxide. Moreover, the effects of OSP on serum PTH and FGF-23 levels and on the expression of NaPi co-transporter proteins in the kidney (NaPi-2a) were examined.

The optimized OSP doses used in this study each contained 0.4 g phosphorus and, as such, correspond to approximately four times the normal daily phosphorus intake in rats (based on a diet containing 0.8% phosphorus and daily food intake of 25 g). Both OSP doses not only caused diarrhea, resulting from its osmotic effect and thus presenting evidence of efficient bowel cleansing, but also resulted in a nearly twofold increase in serum phosphate levels compared to baseline, which is comparable to the range reported in patients. [31,32] Moreover, nephrocalcinosis was induced which was accompanied by an increase in serum creatinine of 1.5 times baseline, indicating the development of AKI (KDIGO). [41] Taken together, these data present a relevant rat model of APN.

The phosphate binder doses used in this study, i.e. a 0.3x molar lanthanum/phosphate ratio and a 0.3x and 1x molar aluminum/phosphate ratio, did not attenuate the acute side-effects of OSP administration. In patients undergoing bowel cleansing, each OSP dose is equivalent to ∼6 g phosphorus (∼18 g phosphate). [12] When the molar ratios of the phosphate binders in this study are extrapolated, this would correspond to a dose of 15 g lanthanum carbonate, and a dose of 4.3 g and 15 g aluminum hydroxide, illustrating that the use of even higher phosphate binder doses to attenuate the acute effects of OSP is not feasible and that further studies, investigating other strategies, are required to prevent phosphate absorption after OSP use to improve its safety profile.

Interestingly, both serum PTH and FGF-23 levels increased after OSP administration. Although well known for PTH and in the setting of hyperphosphatemia in chronic kidney disease (CKD), only scarce data are available on the effect of acute hyperphosphatemia on FGF-23 levels. While in patients with chronic renal failure serum FGF-23 levels have been shown to already increase at the early stages of CKD in the absence of any significant change of serum phosphate or PTH, [44,45] in the present study the increase in serum PTH levels was more pronounced and preceded the rise in serum FGF-23 values. This latter observation is in line with findings of a recent phosphate enema case report, [46] in which it was shown that FGF-23 only peaks after correction of hypocalcemia. This could be a mechanism to avoid reductions in calcitriol which would exacerbate hypocalcemia. [47]

Based on our rather unexpected findings showing no effect of both phosphate binders on the OSP-induced hyperphosphatemia, it was hypothesized that in untreated animals the first OSP dose would decrease NaPi-2b expression, resulting in less phosphate absorption from the second OSP dose as compared to animals receiving phosphate binder treatment. In these latter groups, an amount of phosphate of the first OSP dose would be bound in the intestine, resulting in less suppression of the NaPi-2b transporter and consequently more phosphate absorption from the second OSP dose. This hypothesis was examined by administration of the phosphate binder only with the second OSP dose, so that the first reaction of the intestine towards an increased phosphate load would be the same as in animals only receiving OSP. This strategy, however, also resulted in a twofold increase in serum phosphate levels as compared to baseline (data not shown).

It was observed by others that in the duodenum an adaptive increase in NaPi-2b expression occurs in response to an acute switch from a low-to-high phosphate diet which is accompanied by decreased renal NaPi-2a expression and transient postprandial hyperphosphatemia. [48,49] NaPi-2b mediates the bulk (223C90%) of sodium-dependent intestinal phosphate absorption, [48,50] accounting for ∼40–45% of total intestinal phosphate absorption. This indicates that, in contrast to the kidney, a significant component of intestinal phosphate transport is sodium-independent or occurs in a passive (paracellular) way. [48,51] One can hypothesize that this latter pathway may increase in importance following OSP intake, due to the very high phosphate load and the resulting osmotic effect in the intestinal lumen, which may explain why no significant inhibition of phosphate absorption was observed.

A possible limitation of this study consists in the fact that no urinary parameters were measured. Urine sampling, however, was not feasible as these samples were contaminated with feces (diarrhea) during the housing of the animals in metabolic cages.

In conclusion, a clinically relevant rat model of APN was developed. Animals showed increased serum phosphate and decreased serum calcium levels similar to those reported in humans and developed APN. Serum PTH and FGF-23 levels significantly increased. NaPi-2a expression in the apical membrane of proximal tubules was reduced, confirming the particular involvement of NaPi also in acute phosphate exposure. No evidence was found for an improved safety profile of OSP by using phosphate binders.
